# LncRNA HCG11/miR-579-3p/MDM2 axis modulates malignant biological properties in pancreatic carcinoma via Notch/Hes1 signaling pathway

**DOI:** 10.18632/aging.203167

**Published:** 2021-06-21

**Authors:** Jin Xu, Weixue Xu, Xuan Yang, Zhen Liu, Qinyun Sun

**Affiliations:** 1 Department of Pancreatic and Thyroid Surgery, Shengjing Hospital, China Medical University, Shenyang, China

**Keywords:** pancreatic carcinoma, lncRNA HCG11, ceRNA, malignant behaviors, Notch/Hes1

## Abstract

Background: Increasing reports have revealed that dysregulated expression of long non-coding RNAs (lncRNAs) is involved in pancreatic carcinoma progression. This study intends to explore the function and molecular mechanism of lncRNA HLA complex group 11 (HCG11) in pancreatic carcinoma.

Methods: The expression profiles of HCG11 in pancreatic carcinoma samples were detected by qPCR. Bioinformatics analysis was applied to detect the associations among HCG11/miR-579-3p/MDM2. The malignant properties of pancreatic carcinoma cells were measured by numerous biological assays. Xenograft model was exploited to detect the effect of HCG11 on tumor growth.

Results: A significant increase of HCG11 was occurred in pancreatic carcinoma samples. Knockdown of HCG11 suppressed the progression of pancreatic carcinoma cells. Bioinformatics analysis revealed that HCG11 upregulated MDM2 expression by competitively targeting miR-579-3p. The rescue assays showed that miR-579-3p reversed cell behaviors caused by HCG11, and MDM2 reversed cell properties induced by miR-579-3p. The Notch1 intracellular domain (NICD) and Hes1 protein levels were increased by overexpression of HCG11/MDM2. The tumor growth was suppressed after depletion of HCG11, followed by suppressing Ki67, PCNA and Vimentin expression, increasing TUNEL-positive cells and E-cadherin expression.

Conclusions: Our observations highlighted that HCG11 contributed to the progression of pancreatic carcinoma by promoting growth and aggressiveness, and inhibiting apoptosis via miR-579-3p/MDM2/Notch/Hes1 axis.

## INTRODUCTION

Pancreatic carcinoma is one of the most lethal malignant tumor in the digestive system, with 216,000 new cancer cases worldwide every year, causing more than 200,000 deaths each year [1, 2]. Advances in early diagnosis, surgical excision, systematic chemotherapy and targeted therapy have improved the therapy of pancreatic carcinoma, but have not translated into practical values, with a lower survival rate for patients with pancreatic carcinoma [3–5]. The miserable ending of patients with pancreatic carcinoma underscores the importance of expounding the molecular mechanisms of pancreatic carcinoma progression, which are not yet understood. Therefore, the identification of new diagnostic and prognostic biomarkers related to the progression of pancreatic carcinoma has important clinical relevance and significance.

Previous studies have revealed that nearly 98% of human genome transcripts are non-coding RNAs (ncRNAs), which have no protein coding ability [6]. Long non coding RNAs, more than 200 nt in length, are dominant in the ncRNA family [7]. Abnormal expression of lncRNAs has been discovered to have crucial roles in gene control as well as might act as an oncogene or tumor suppressor in cancer progression [8], including pancreatic carcinoma [9]. For example, linc00514 acted as an oncogene in pancreatic carcinoma to promote the tumor progression [10]. LncRNA CYTOR can promote the development of pancreatic carcinoma by increasing the cell proliferation and migration [11]. Besides that, lncRNAs can serve as competing endogenous (ceRNAs) or RNA sponges, which targeting microRNAs (miRNAs) to sequester them and arrest their effect on target mRNAs [12, 13]. This is, lncRNAs and mRNAs can influence each other by competitively binding with a miRNA response element (MRE) to affect post-transcriptional modulation [14]. For example, LINC01559 acted as a ceRNA of miR-1343-3p to upregulate RAF1, thus promoting the progression of pancreatic carcinoma [15]. Also, lncRNA OIP5-AS1 accelerated the malignancy of pancreatic carcinoma in regulation of miR-429/FOXD1 [16]. All these findings underscored the importance of lncRNAs in the development of pancreatic carcinoma.

LncRNA HLA complex group 11 (HCG11), as a common lncRNA, has been identified in various cancers [17]. In hepatocellular carcinoma, HCG11 suppressed tumor cells apoptosis to promote the progression of hepatocellular carcinoma [18]. Besides, HCG11 has been reported to increase the cells growth and mobility in gastric cancer by modulating of miR-1276/CTNNB1 [17]. Inversely, in glioma, HCG11 has been illustrated to limit the development of glioma in regulation of miR-496/CPEB3 axis [19]. Moreover, HCG11 also inhibited the progression of laryngeal carcinoma by regulation of miR-4469/APOM [20]. Analysis from the above published literature revealed that HCG11 may play a dual role in different cancers, which highlighted the function of HCG11 in cancer progression. However, the expression and mechanism of HCG11 in pancreatic carcinoma remain not elucidated.

Therefore, in our study, the expression of HCG11 and its potential target miR-579-3p/MDM2 were analyzed in clinical pancreatic carcinoma and corresponding para-carcinoma tissues. Moreover, the biological function of HCG11/miR-579-3p/MDM2 in pancreatic carcinoma was analyzed by *in vitro* and *in vivo* models.

## RESULTS

### Expression profiles of HCG11 in pancreatic carcinoma tissues and cells

**Figure 1:**
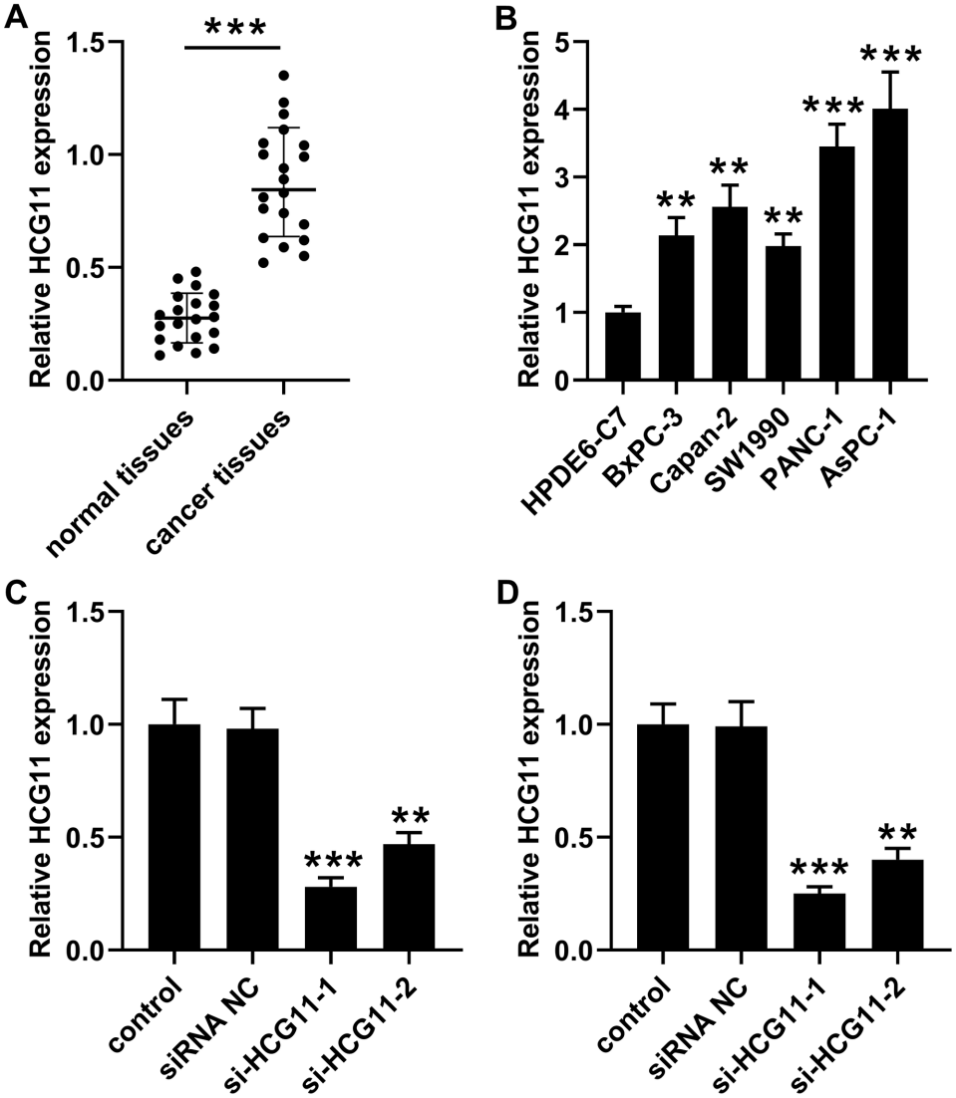
**High expression of HCG11 was observed in pancreatic carcinoma tissues and cells.** (**A**) qPCR assay was performed to detect HCG11 expression in 20 pairs of pancreatic carcinoma tissues and corresponding normal tissues. ^***^p<0.001 vs. normal tissues. (**B**) HCG11 expression in pancreatic carcinoma cell lines (BxPC-3, Capan-2, SW1990, PANC-1 and AsPC-1) was also detected by qPCR. ^**^p<0.01, ^***^p<0.001 vs. HPDE6-C7. (**C**, **D**) With transfected si-HCG11-1 or si-HCG11-2 in PANC-1 and AsPC-1 cells, qPCR was applied to detect HCG11 expression. ^**^p<0.01, ^***^p<0.001 vs. siRNA NC.

To identify the expression level of HCG11 in pancreatic carcinoma, qPCR experiment was utilized to detect the expression of HCG11 in 20 pairs of pancreatic carcinoma tissues. Significantly, a marked augmentation of HCG11 expression was observed in pancreatic carcinoma tissues when compared with the corresponding non-cancerous tissues (Figure 1A, *p*<0.001). Next, we performed qPCR to detect HCG11 expression in various pancreatic carcinoma cancer cell lines. Compared with HPDE6-C7 cells, we observed that HCG11 expression was highly increased in pancreatic carcinoma cells including BxPC-3, Capan-2, SW1990, PANC-1 and AsPC-1 (Figure 1B, *p*<0.001). Due to the high expression rates of HCG11 in PANC-1 and AsPC-1 cells, these two cells were used for subsequent experiments.

### Knockdown of HCG11 inhibited growth, movement and caused apoptosis in pancreatic carcinoma cells

**Figure 2:**
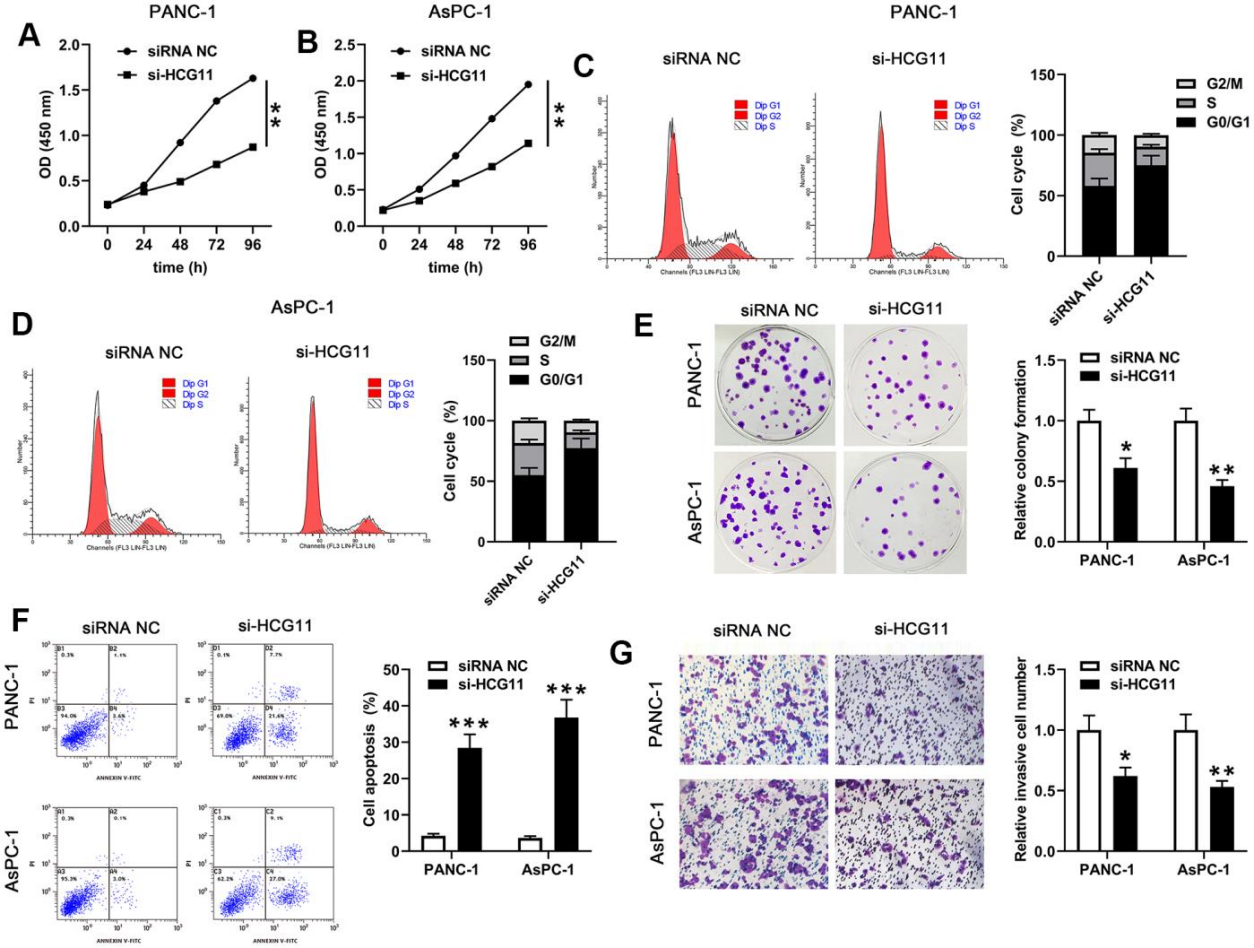
**HCG11 depletion suppressed proliferation, cycle, colony formation, mobility of PANC-1 and AsPC-1 cells but promoted their apoptosis.** (**A**, **B**) CCK-8 assay was used to examine the viability of PANC-1 and AsPC-1 cells after transfected with si-HCG11. (**C**, **D**) After transfection with si-HCG11, flow cytometry assay was performed to detect the cell cycle of PANC-1 and AsPC-1 cells. (**E**) Plate clone formation assay was conducted to measure the number of cell clones in PANC-1 and AsPC-1 cells. (**F**) After knockdown of HCG11, flow cytometry assay was applied to detect the apoptotic rates in PANC-1 and AsPC-1 cells. (**G**) Transwell assay was used to determine the invaded number of PANC-1 and AsPC-1 cells after knockdown of HCG11. ^*^p<0.05, ^**^p<0.01, ^***^p<0.0001 vs. siRNA NC.

To examine the effect of HCG11 in pancreatic carcinoma, si-HCG11-1 or si-HCG11-2 was applied to knockdown of HCG11 in pancreatic carcinoma cells. Both si-HCG11-1 and si-HCG11-2 significantly reduced the expression of HCG11, but si-HCG11-1 was more efficient, so it was selected for subsequent experiments (Figure 1C, 1D, *p*<0.01). After knockdown of HCG11, we observed that the OD values of pancreatic carcinoma cells were decreased significantly (Figure 2A, 2B, *p*<0.01). Moreover, the data from flow cytometry assay indicated that suppression of HCG11 in pancreatic carcinoma cells controlled the cell cycle by inducing G0/G1 arrest compared with the siRNA NC group (Figure 2C, 2D). Besides, we also discovered that depletion of HCG11 notably reduced the number of clones in pancreatic carcinoma cells (Figure 2E, *p*<0.05). The results insinuated that depletion of HCG11 suppressed the growth of pancreatic carcinoma cells. Thereafter, an apoptosis experiment was conducted to detect the effect of HCG11 on pancreatic carcinoma cells apoptosis. As expected, the apoptotic percentage of pancreatic carcinoma cells was significantly increased after knockdown of HCG11 (Figure 2F, *p*<0.0001). Besides, we also detected that depletion of HCG11 reduced the invaded number of pancreatic carcinoma cells (Figure 2G, *p*<0.05). All these observations illustrated that HCG11 played an active role in the progression of pancreatic carcinoma.

### HCG11 acted as a ceRNA by direct sponging of miR-579-5p

**Figure 3:**
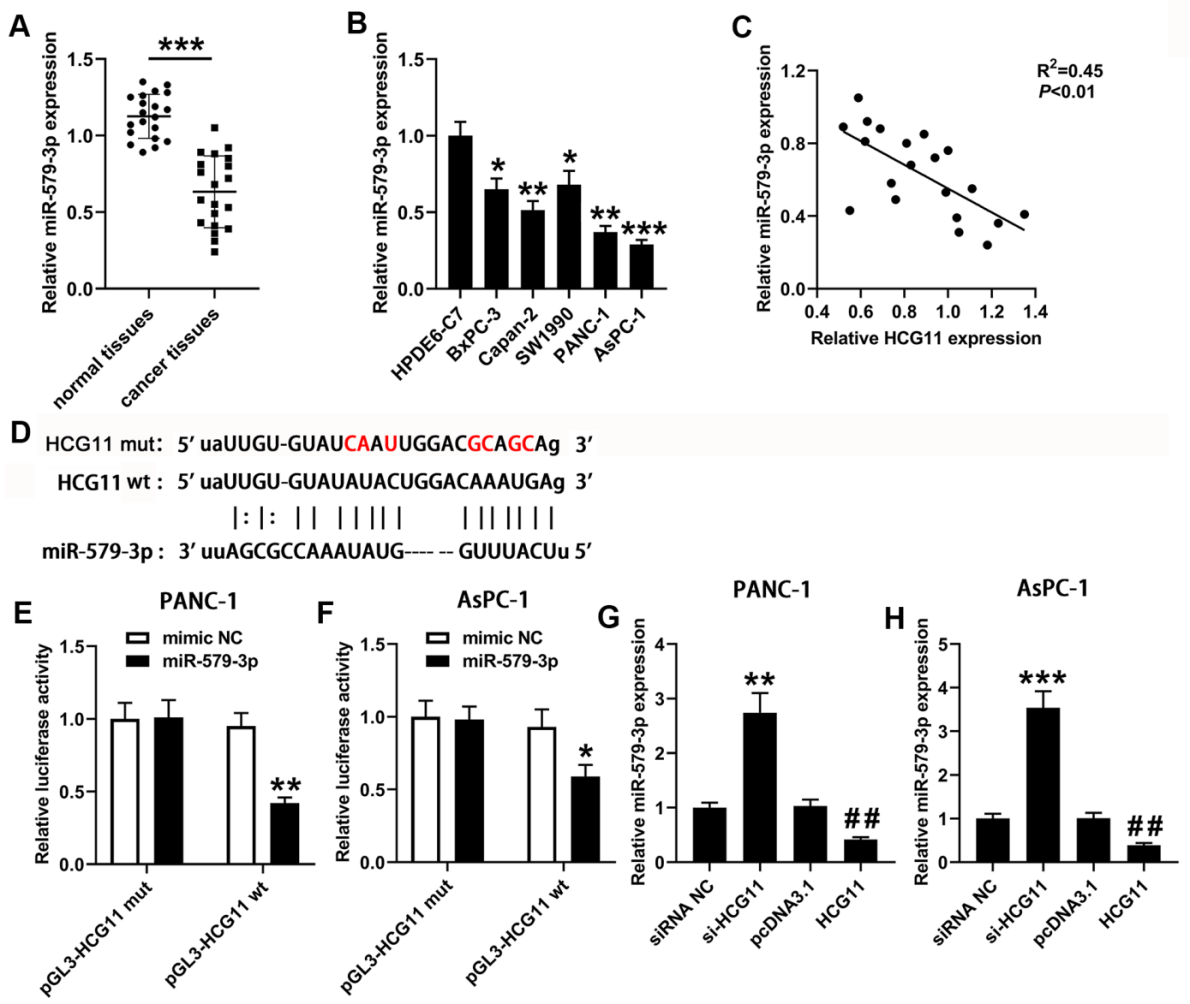
**HCG11 acted as a sponge for miR-579-3p in pancreatic carcinoma cells.** (**A**) qPCR was performed to measure miR-579-3p expression in 20 pairs of pancreatic carcinoma tissues and corresponding normal tissues. ^***^p<0.001 vs. normal tissues. (**B**) miR-579-3p expression was also detected in pancreatic carcinoma cell lines (BxPC-3, Capan-2, SW1990, PANC-1 and AsPC-1) by qPCR. ^*^p<0.05, ^**^p<0.01, ^***^p<0.001 vs. HPDE6-C7. (**C**) Data from qPCR presented that a negative association was observed in HCG11 expression and miR-579-3p expression. ^**^p<0.01. (**D**) The targeting sequences between HCG11 and miR-579-3p were presented. (**E**, **F**) The luciferase activity in PANC-1 and AsPC-1 cells was measured by the luciferase reporter assay after transfected with miR-579-3p mimic/NC and HCG11-mut/wt vector. (**G**, **H**) qPCR assay was used to detect miR-579-3p expression in pancreatic carcinoma cell lines with up or down regulation of HCG11. ^**^p<0.01, ^***^p<0.001 vs. siRNA NC, ^##^*p*<0.01 vs. pcDNA3.1.

A plenty of reports presented that lncRNAs can serve as ceRNAs, eliminating the endogenous inhibitory effect of these miRNAs on their targeted transcripts. Then, we used the bioinformatics software starBase v2.0 (http://starbase.sysu.edu.cn/) to explore the miRNAs that targeted with HCG11. The data showed an underlying combination of HCG11 and miR-579-3p, the predicted targeting sequences were presented in Figure 3D. To further identify the interaction, the HCG11 sequences including the wt or mut miR-579-3p targeting sites was inserted into the downstream of pGL-3 vector. Next, the function of miR-579-3p mimic on the luciferase activity was observed. In PANC-1 and AsPC-1 cells, the luciferase activity in wt-pGL3-HCG11 group was obviously reduced than the control group after miR-579-3p mimic treatment (Figure 3E, 3F, *p*<0.05). Nonetheless, no obvious change of luciferase activity has been observed in mut-pGL3-HCG11 group when treated by miR-579-3p mimic. Data from Figure 3A, 3B showed that miR-579-3p expression was obviously decreased in pancreatic carcinoma samples when compared with the adjacent normal tissues and HPDE6-C7 cells. Moreover, miR-579-3p expression was significantly negatively correlated with the expression of HCG11 (Figure 3C, *p*<0.01). Additionally, the qPCR results from PANC-1 and AsPC-1 cells showed that depletion of HCG11 increased miR-579-3p expression (Figure 3G, 3H, *p*<0.01). Inversely, overexpression of HCG11 obviously reduced miR-579-3p expression (Figure 3G, 3H, *p*<0.01), further validating the interaction between HCG11 and miR-579-3p.

### MDM2 was validated as a downstream target of miR-579-3p and was co-modulated by HCG11/miR-579-3p

**Figure 4:**
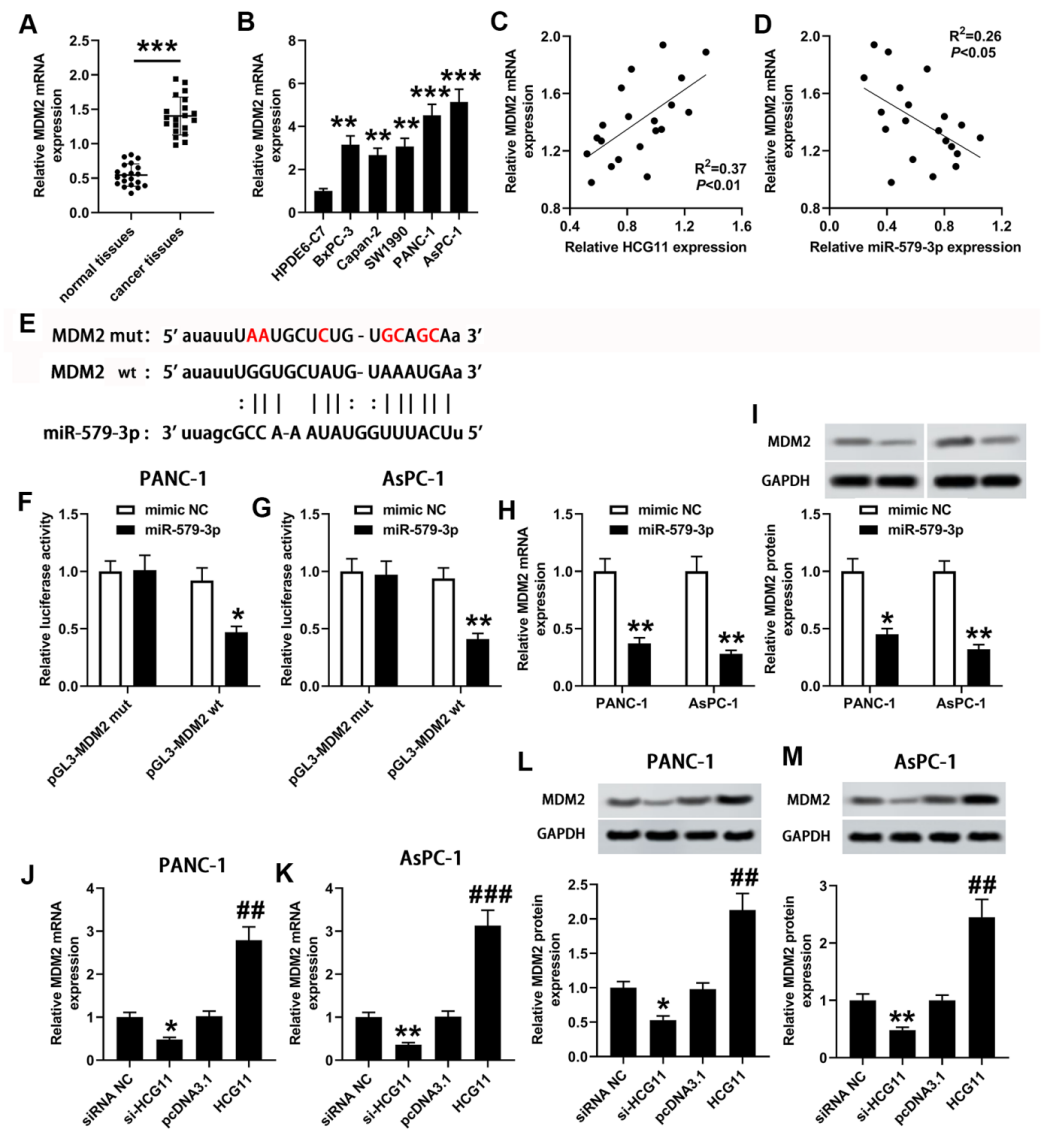
**miR-579-3p directly targeted MDM2 to suppress MDM2 expression.** (**A**) qPCR assay was used to examine MDM2 expression in 20 pairs of pancreatic carcinoma tissues and corresponding normal tissues. ^***^p<0.001 vs. normal tissues. (**B**) MDM2 expression in pancreatic carcinoma cell lines (BxPC-3, Capan-2, SW1990, PANC-1 and AsPC-1) was also detected by qPCR assay. ^**^p<0.01, ^***^p<0.001 vs. HPDE6-C7. (**C**, **D**) Analysis from qPCR revealed that MDM2 expression was positively related to HCG11 expression, and negatively associated with miR-579-3p expression. (**E**) The binding sites between miR-579-3p and MDM2 were exhibited. (**F**, **G**) The luciferase activity in PANC-1 and AsPC-1 cells was measured by the luciferase reporter assay after transfected with miR-579-3p mimic/NC and MDM2-mut/wt vector. (**H**, **I**) MDM2 mRNA and protein expression levels in pancreatic carcinoma cells were detected by qPCR and western blotting assays when treated with miR-579-3p mimic or NC. ^*^p<0.05, ^**^p<0.01 vs. mimic NC. (**J**–**M**) MDM2 mRNA and protein expression levels in pancreatic carcinoma cells were detected by qPCR and western blotting assays when treated with siRNA NC, si-HCG11, pcDNA3.1, and pcDNA3.1-HCG11. ^*^p<0.05, ^**^p<0.01 vs. siRNA NC, ^##^*p*<0.01, ^###^*p*<0.001 vs. pcDNA3.1.

To investigate the presumptive target genes of miR-579-3p, we used Targetscan to search for the candidate genes. Bioinformatics analysis revealed that miR-579-3p directly binds MDM2 (Figure 4E). To further affirm the association between miR-579-3p and MDM2, a wt or mut pGL3-MDM2 vector was generated, and wt or mut pGL3-MDM2 was co-transfected with miR-579-3p mimic or NC into PANC-1 and AsPC-1 cells. Compared with the corresponding control group, the luciferase activity was notably decreased in cells that co-transfected with wt pGL3-MDM2 luciferase vector and miR-579-3p mimic (Figure 4F, 4G, *p*<0.05). However, the inhibitory effect was eliminated when pGL3-MDM2 vector that included mut binding sequences were co-transfected with miR-579-3p mimic (Figure 4F, 4G, *p*<0.05). Next, the qPCR was conducted to analyze MDM2 expression in pancreatic carcinoma samples, indicating that a marked augmentation of MDM2 was presented in pancreatic carcinoma samples when in contrast with the adjacent samples and HPDE6-C7 cells (Figure 4A, 4B, *p*<0.01). MDM2 expression was actively related to the expression of HCG11 (Figure 4C, *p*<0.01). Inversely, MDM2 expression was negatively correlated with miR-579-3p (Figure 4D, *p*<0.05). Importantly, we also discovered that MDM2 expression both at mRNA and protein levels were obviously decreased in PANC-1 and AsPC-1 cells when miR-579-3p mimic treatment (Figure 4H, 4I), further confirming the interaction between miR-579-3p and MDM2. Also, we analyzed the influence of HCG11 on MDM2 expression. As shown in Figure 4J–4M, depletion of HCG11 reduced MDM2 expression at transcription and translation levels in pancreatic carcinoma cells. However, MDM2 expression were obviously increased in PANC-1 and AsPC-1 cells at both mRNA and protein levels when upregulation of HCG11. These results suggested that MDM2 was affirmed as a target of miR-579-3p and regulated by HCG11/miR-579-3p, insinuating that HCG11, miR-579-3p and MDM2 may establish a ceRNA network to regulate the progression of pancreatic carcinoma.

### HCG11 promoted the biological behaviors of pancreatic carcinoma cells by targeting miR-579-3p/MDM2 axis and activating Notch/Hes1 pathway

**Figure 5:**
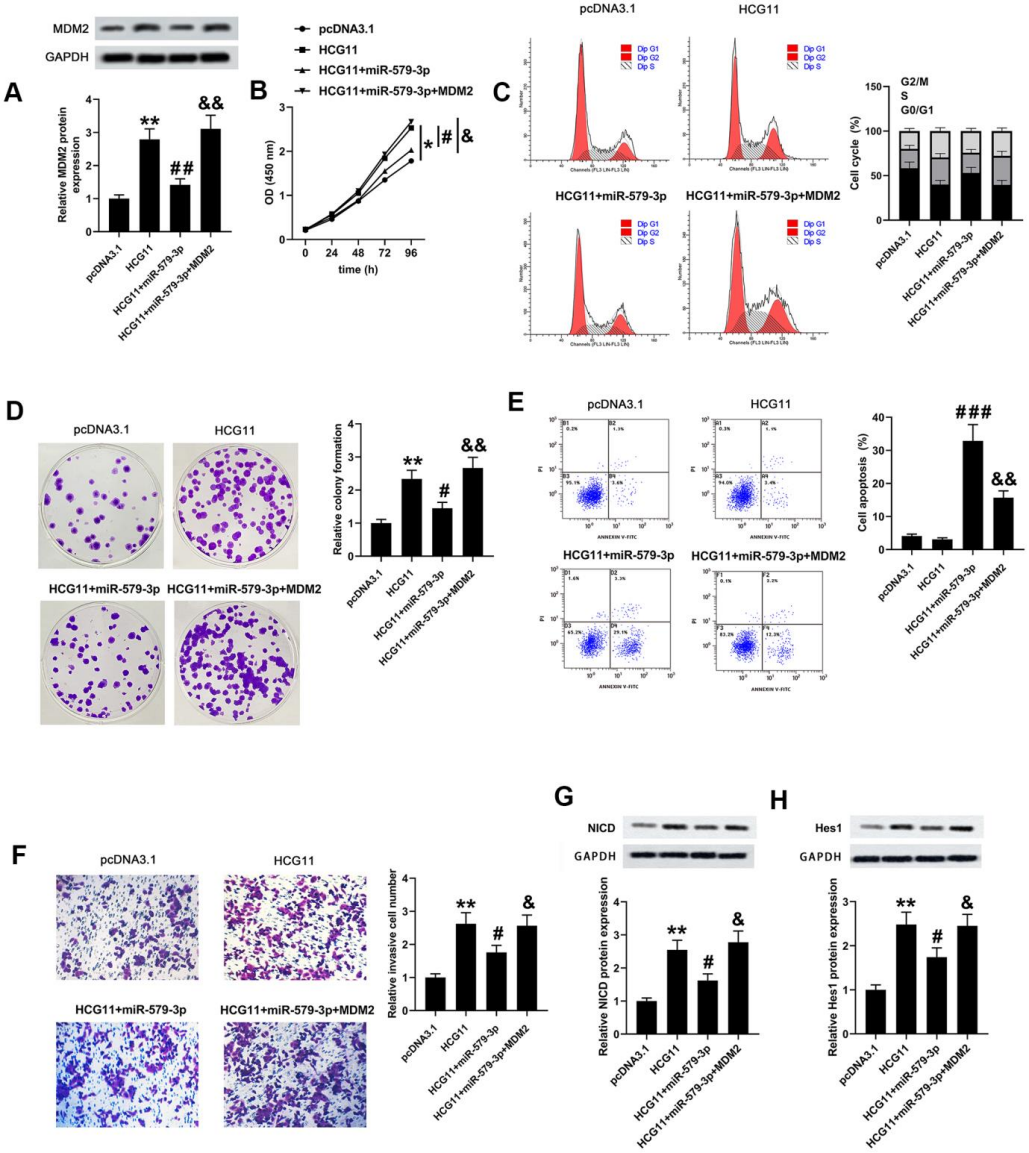
**HCG11, miR-579-3p and MDM2 co-regulated the biological behaviors of pancreatic carcinoma cells partly by regulating Notch/Hes1 pathway.** (**A**) MDM2 protein expression AsPC-1 cells was detected by western blotting assay after upregulation of HCG11, HCG11+miR-579-3p, or HCG11+miR-579-3p+MDM2. (**B**–**D**) The OD values, cell cycle and clones number in AsPC-1 cells were analyzed by CCK-8, flow cytometry and colony formation assays when treatment with HCG11, HCG11+miR-579-3p, or HCG11+miR-579-3p+MDM2. (**E**) The apoptosis rates of AsPC-1 cells were determined by flow cytometry assay following HCG11, HCG11+miR-579-3p, or HCG11+miR-579-3p+MDM2 treatment. (**F**) The invaded number of AsPC-1 cells was estimated by Transwell chamber when treated by HCG11, HCG11+miR-579-3p, or HCG11+miR-579-3p+MDM2. (**G**, **H**) The protein expression levels of NICD and Hes1 in AsPC-1 cells were measured by western blotting assay after upregulation of HCG11, HCG11+miR-579-3p, or HCG11+miR-579-3p+MDM2. ^*^p<0.05, ^**^p<0.01 vs. pcDNA3.1, ^#^*p*<0.05, ^##^*p*<0.01, ^###^*p*<0.001 vs. HCG11, ^&^*p*<0.05, ^&&^*p*<0.01 vs. HCG11+miR-579-3p.

To validate the network among HCG11, miR-579-3p and MDM2, we carried out a series of biological experiments to observe the effect of HCG11/miR-579-3p/MDM2 on AsPC-1 cells growth, apoptosis and mobility. Firstly, we observed that MDM2 expression was co-regulated by HCG11, miR-579-3p and MDM2 (Figure 5A, *p*<0.01). Then, data from CCK-8, flow cytometry, plate cloning formation and Transwell assays suggested that upregulation of miR-579-3p suppressed the pro-oncogenic effect of HCG11 in pancreatic carcinoma cells. However, the above results were inversed by MDM2 upregulation (Figure 5B–5F, *p*<0.05). These data revealed that the pro-oncogenic effect of HCG11 in pancreatic carcinoma was achieved by targeting miR-579-3p to upregulate MDM2. Thereafter, the protein expression levels of NICD and Hes1 were also examined. We discovered that upregulation of HCG11 promoted the expression of NICD and Hes1, which can be eliminated by miR-579-3p overexpression. However, the effect of miR-579-3p on NICD and Hes1 expression was rescued by MDM2 expression (Figure 5G, 5H, *p*<0.05). These data insinuated that HCG11/miR-579-3p/MDM2 functioned as a ceRNA network to promote the progression of pancreatic carcinoma cells by inactivating Notch/Hes1 pathway.

### Knockdown of HCG11 inhibited the tumor growth by regulating a range of biological behaviors

**Figure 6:**
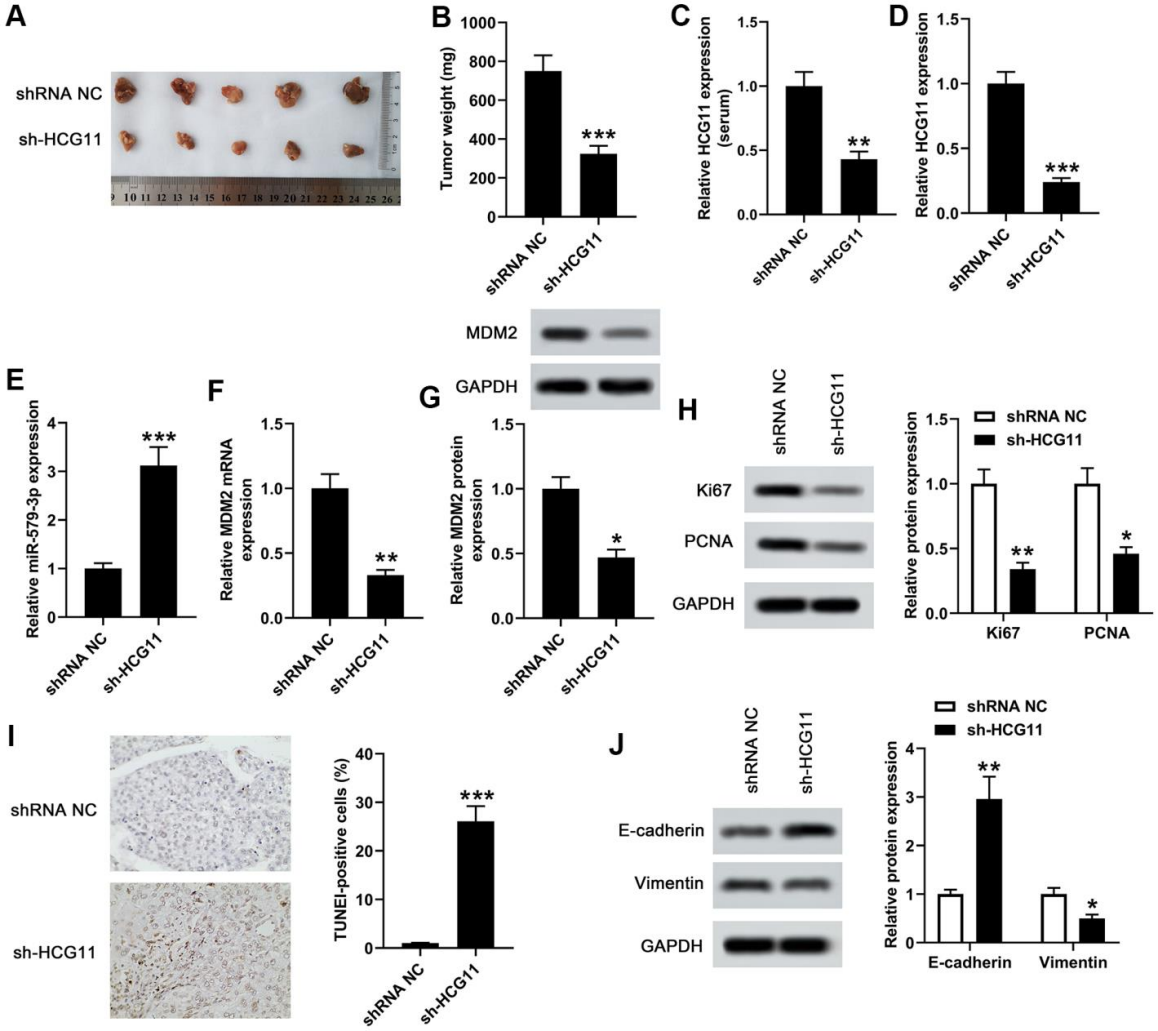
**Knockdown of HCG11 inhibited the tumor growth in xenograft mouse model.** (**A**) We examined the effect of knockdown of HCG11 on the regulation of subcutaneous transplantation mouse model of pancreatic carcinoma by using AsPC-1 cells. (**B**) Four weeks later, the tumor weight of sh-HCG11 group and shRNA NC group was measured. (**C**) qPCR assay was applied to detect HCG11 expression (serum) in sh-HCG11 group and shRNA NC group. (**D**) HCG11 expression (tissues) in sh-HCG11 group and shRNA NC group was also detected by qPCR assay. (**E**) The expression level of miR-579-3p (tissues) in sh-HCG11 group and shRNA NC group was measured by qPCR. (**F**, **G**) The mRNA and protein levels of MDM2 (tissues) in sh-HCG11 group and shRNA NC group were measured by qPCR and western blotting assays. (**H**) The levels of proliferation associated proteins including Ki67 and PCNA were detected by western blotting assay in sh-HCG11 group and shRNA NC group. (**I**) The TUNEL-positive cells in sh-HCG11 group and shRNA NC group were measured by TUNEL assay. (**J**) Western blotting assay was applied to detect E-cadherin and Vimentin expression in sh-HCG11 group and shRNA NC group. ^*^p<0.05, ^**^p<0.01, ^**^p<0.001 vs. shRNA NC.

To investigate the function of HCG11 depletion in the modulation of tumor growth, we detect the effect of HCG11 using AsPC-1 cells subcutaneous xenograft mouse model. After injection, the tumor volumes were tested every 7 days for 4 weeks. We observed that knockdown of HCG11 notably suppressed tumor growth (Figure 6A). The tumor weight of AsPC-1-HCG11-knockdown (KD) group was obviously lower than the control group (Figure 6B, *p*<0.001). Besides, we also observed a decrease of HCG11 expression in the serum of sh-HCG11 nude mice (Figure 6C, *p<0.01*). Moreover, the decreased HCG11 expression and the increased miR-579-3p expression were observed in HCG11-KD tumors (Figure 6D, 6E, *p*<0.001). Besides, the mRNA and protein levels of MDM2 reduced in HCG11-KD tumors (Figure 6F, 6G, *p*<0.05). Furthermore, the proliferation associated genes Ki67 and PCNA were obviously decreased in HCG11-KD tumors (Figure 6H, *p*<0.05). As well, the TUNEL-positive cells in HCG11-KD tumors showed significant changes, accompanied by obvious increase (Figure 6I, *p*<0.001). Additionally, we also discovered that in HCG11-KD tumors, E-cadherin protein level increased, while Vimentin level decreased (Figure 6J, *p*<0.05). All these results insinuated that depletion of HCG11 suppressed the tumor growth by targeting miR-579-3p/MDM2, thereby inhibiting tumor cells proliferation, promoting tumor cells apoptosis and blocking tumor cells movement.

## DISCUSSION

An increasing number of lncRNAs have been verified to be biomarkers in pancreatic carcinoma development. For example, lncRNA TUSC7 served as a tumor suppressor in pancreatic carcinoma through regulating miR-571a-5p expression [21]; lncRNA MACC1-AS1 accelerated the development of pancreatic carcinoma by activating PAX8/NOTCH1 pathway [22]; lncRNA PVT1 acted as an oncogenic lncRNA in pancreatic carcinoma by sponging miR-488 [23]. All these reports indicated the importance function of lncRNAs in modulating mRNA expression and cell characteristic in human tumors. In this study, we centered on exploring the effect of HCG11 in pancreatic carcinoma. A marked augmentation of HCG11 was first discovered in both pancreatic carcinoma tissues and cell lines. Functionally, we also discovered that depletion of HCG11 notably limited cell growth, blocked cell cycle, induced cell apoptosis and suppressed cell mobility. Additionally, we also illustrated that depletion of HCG11 inhibited the tumor growth *in vivo*. So, all these observations insinuated that HCG11 acted as an oncogenic lncRNA in pancreatic carcinoma progression.

Recently, increasing evidence pointed out that lncRNAs primarily functioned as a miRNA sponge to play their post-transcriptional roles as ceRNAs, which was more significant than the traditional anti-miRNA method [24]. MiRNAs, the most widely researched non-coding RNAs, functioned as oncogenes or tumor suppressors to regulate tumor initiation and progression [25]. In our study, by applying bioinformatics analysis, we found several miRNAs potentially bind with HCG11, among which miR-579-3p was negatively regulated by HCG11 and was significantly downregulated in pancreatic carcinoma tissues and cells. Moreover, the function of miR-579-3p has been revealed in several tumors. For example, the suppression of lung squamous cancer cell invasion and migration by miR-579-3p, was previously reported by Wu and colleagues [26]. Besides, miR-579-3p expression was discovered to be declined and to control the progression of melanoma [27]. Therefore, miR-579-3p was identified as a specific target of HCG11. Herein, low expression of miR-579-3p was observed in pancreatic carcinoma tissues and cells, consistent with the findings in melanoma and lung squamous cell carcinoma [26, 27], as well as negatively associated with the expression of HCG11. Moreover, we also discovered that upregulation of miR-579-3p inhibited cell growth, induced cell cycle arrest, promoted cell apoptosis and suppressed cell mobility. All these findings insinuated that miR-579-3p exerted tumor suppressor function in pancreatic carcinoma. Additionally, to explore the effect of miR-579-3p over-expression on the malignant behavior of AsPC-1 cells and on the tumor-promoting effect of HCG11, we artificially over-expressed miR-579-3p and HCG11 in AsPC-1 cells after reviewing relevant literature [19, 28–30]. After over-expression of HCG11, the malignant behaviors of cells were strengthened, and those behaviors were inhibited after over-expression of miR-579-3p, which can better illustrate the importance of miR-579-3p in AsPC-1 and its influence on HCG11.

It has been widely documented that lncRNA-miRNA-mRNA interactions exert an important function in tumorigenesis [31]. Bioinformatics and luciferase assays indicated that miR-579-3p targeted to 3’UTR of MDM2 and inhibited MDM2 expression at both mRNA and protein levels. MDM2 upregulation was observed in numerous human tumors, including pancreatic carcinoma [32]. Additionally, previous studies have revealed that MDM2 was identified as one of the primary genes that accelerate the metastasis of pancreatic carcinoma [32, 33]. Importantly, our PCR results presented that MDM2 expression in pancreatic carcinoma samples was notably increased. Moreover, MDM2 expression was positively related to HCG11 expression and negatively associated with miR-579-3p expression. Additionally, consistent with the findings from Shi et al. [32], we also discovered that upregulation of MDM2 promoted cell growth, relieved cell cycle arrest, suppressed cell apoptosis and increased cell mobility, which further confirming the carcinogenic effect of MDM2 in pancreatic carcinoma.

The highly-conserved Notch signaling pathway plays numerous essential roles in the development of various cells, tissues and organs from *Drosophila* to humans, and dysregulated Notch signaling pathway contributes to some disorders, such as vascular and bone defects, as well as some tumors [34]. For example, a recent study revealed that the Notch signaling pathway was activated in the progression of pancreatic ductal adenocarcinoma [35] and another study has also proposed that overexpression of Notch1 promoted the development of pancreatic cancer [36], which highlighting the importance of Notch signaling pathway in pancreatic cancer. When Notch ligands, including Jagged (JAG)1, JAG2M delta like canonical notch ligand (DLL)1, DLL3 and DLL4, interact with Notch transmembrane receptors, this binding causes the cleavage of Notch receptor by proteases to release NICD [37]. Then, NICD travels to the nucleus and binds to DNA binding proteins to assemble a transcription complex that activates downstream target genes such as Hes1 [37]. In our study, we observed that overexpression of HCG11 in AsPC-1 cells could increase NICD and Hes1 expression. Besides, we also discovered that NICD and Hes1 expression was promoted by HCG11 upregulation or suppressed by miR-579-3p overexpression. A recent study by Luo et al. showed that MDM2 inhibition improved cisplatin-induced renal injury in mice by inactivation of Notch/Hes1 signaling pathway [38]. Similarly, our study discovered that overexpression of MDM2 increased the levels of NICD and Hes1. In a word, our study revealed that HCG11/miR-579-3p/MDM2 acted as a ceRNA network to promote the progression of pancreatic carcinoma by activating Notch/Hes1 pathway, further affirming the importance of MDM2/Notch/Hes1 in pancreatic carcinoma.

In conclusion, the pivotal observations in this study illustrated that HCG11 increased the expression of MDM2 via competitively targeting miR-579-3p to promote the Notch/Hes1 pathway, thereby promoting the progression of pancreatic carcinoma. We speculated that HCG11/miR-579-3p/MDM2 axis could be an underlying therapeutic target in the treatment of pancreatic carcinoma.

## MATERIALS AND METHODS

### Human samples

Twenty pairs of surgical samples including pancreatic carcinoma tissues and normal pancreatic tissues were originally taken from patients in our hospital. Surgical samples were gathered for the calculation of HCG11, miR-579-3p and MDM2 expression. The protocol of this study was performed based on the Declaration of Helsinki and has been approved by the Ethics Committee of our hospital. Written informed consent was achieved from each participant.

### Cell lines and culture

Pancreatic carcinoma cell lines including BxPC-3, SW1990, PANC-1, AsPC-1, Capan-2 were obtained from the American Type Culture Collection (ATCC; Manassas, USA). Human immortalized pancreatic duct epithelial cells (HPDE6-C7) were afforded by Shanghai Cell Bank of the Chinese Academy of Sciences (Shanghai, China). All cells were grown in DMEM including 10% fetal bovine serum (FBS) in an incubator (37° C, 5% CO_2)_.

### Cell transfection and treatment

Small fragments including si-HCG11-1, si-HCG11-2, si-RNA negative control (NC), pcDNA3.1-vector, pcDNA3.1-HCG11, pcDNA3.1-MDM2, miR-579-3p mimic and NC were designed and synthesized by Sangon (Shanghai, China). Then, the fragments were transfected into cells by using Lipofectamine 2000 to regulate the expression of HCG11, miR-579-3p and MDM2, respectively.

### RNA isolation and qRT-PCR

TRIzol solution (TaKaRa, Japan) was performed to isolate RNA from pancreatic carcinoma samples. Then, cDNA was formed with a reverse transcription kit following the supplier’s specification. Then, the ABI Prism 7700 sequence detection system was applied to perform the qRT-PCR process with the subsequent steps: 95° C for 5 min, 40 cycles of 95° C for 30 s, 60° C for 45 s, and 72° C for 90 s and 72° C for 10 min. The relative expression of HCG11, miR-579-3p and MDM2 were analyzed by 2^-ΔΔCT^ method. GAPDH was deemed as the standard control for detection of HCG11 and MDM2. U6 was deemed as the standard control for determination of miR-579-3p. The primers were listed as below:

HCG11, F: 5’-AATGGTGGTAGGAGGGAGGA-3’,

R: 5’-CACACAGGGGAATGAAGAGG-3’;

MiR-579-3p, F: 5’-GCACGGAACTTCCCTTGACGTC-3’,

R: 5’-GCTCTAGGGATCGTCGCCGAA-3’;

MDM2, F: 5’-ATGTGCAATACCAACATCTCTGTGTC-3’,

R: 5’-GCTGACTTACAGCCACTAAATTTC-3’;

GAPDH, F: 5’-CGGAGTCAACGGATTTGGTCGTAT-3’,

R: 5’-AGCCTTCTCCATGGTGGTGAAGAC-3’;

U6, F: 5’-CGCTTCGGCAGCACATATACTAA-3’,

R: 5’-TATGGAACGCTTCACGAATTTGC-3’.

### Cell viability and colony formation assays

The viability of PANC-1 or AsPC-1 cells was estimated with the support of cell counting kit-8 (CCK-8). Briefly, the cells were implanted in a 96-well plate with a concentration of 5×10^3^ cells/well and treated with si-HCG11 or siRNA NC. Cell viability was analyzed with the assistance of CCK-8 kit (Beyotime, China) based on the supplier’s introduction. Each experiment was independently repeated three times.

For colony formation assay, 500 PANC-1 or AsPC-1 cells were implanted into a 60 mm dish, transfected with si-HCG11 or siRNA NC, and grew in medium, which replaced every three days. After 14 days, visible colonies that had been fixed were counted.

### Flow cytometr*y*

After treatment, the cell density was adjusted to 1×10^6^ cells/mL. Next, 1 mL of the cell suspension were centrifuged and the sediment was placed in the pre-cooling 70% ethanol for fixing at 4° C overnight. Subsequently, 100 μL of cell suspension was put into 50 μg of propidine iodide (PI) including RNAase and left to react for 30 min away from the light. Finally, the percentage of the cells in G0-G1, S and G2-M phases were counted and compared at 488 nm by a flow cytometry.

The apoptotic rate of cells was tested by Annexin V-FITC/PI double staining. Briefly, the cells were collected by trypsinization and rinsed with pre-cooling phosphate buffer saline (PBS). Next, the cells were cultured with Annexin V-FITC and PI for 15 min in the dark. In the end, the apoptosis rate was estimated at 488 nm by a flow cytometry.

### Western blotting

Protein was extracted from pancreatic carcinoma samples and quantified by bicinchoninic acid (BCA) method. Next, total protein was isolated by SDS-PAGE and blotted onto PVDF membranes. Then, the membranes and contents were probed with the primary antibodies against MDM2 (#86934, 1:2000, CST), Ki67 (#9449, 1:1000, CST), NICD (ab128076, 1:1000, Abcam), Hes1 (ab71559, 1:1000, Abcam), PCNA (#2586, 1:2000, CST), E-cadherin (#14472, 1:2000, CST), Vimentin (#5741, 1:2000, CST) and GAPDH (#5174, 1:2000, CST) at 4° C overnight. After blotted with the secondary antibody, the proteins were developed by enhanced chemiluminescence and visualized by Image J software.

### Dual luciferase reporter gene assay

Bioinformatics software predicted that the targeted association between HCG11 and miR-579-5p, as well as miR-579-5p and MDM2 were existed. The luciferase assay was conducted in PANC-1 and AsPC-1 cells to further confirm the association among HCG11, miR-579-5p and MDM2. Firstly, the corresponding plasmids (including wild type (wt)-pGL3-HCG11, mutant (mut)-pGL3-HCG11, wt-pGL3-MDM2 and mut-pGL3-MDM2) and miR-579-3p mimic/NC were co-transfected into cells with the assistance of Lipofectamine 2000 reagent. After incubation for 48 h, the dual-luciferase reporter assay system was carried out to analyze the luciferase activity.

### Cell invasion assay

Cell invasion assay was implemented utilizing Transwell chambers (Corning, USA). After the matrigel (BD Biosciences, USA) was pre-coated in the upper chamber, PANC-1 or AsPC-1 cells (200 μL, 3×10^5^ cells/well) were implanted into the above chamber with medium excluding serum. Then, DMEM medium including 10% FBS was filled into the under chamber, which was placed in 37° C incubator with 5% CO_2_ for 24 h. Finally, the stained cells were counted under a microscope to estimate the invasion capacity.

### TdT-mediated dUTP-FITC nick end-labeling (TUNEL) assay

The apoptosis of pancreas was measured through TUNEL assay utilizing a commercially available *In Situ* Cell Death Detection Kit (Roche Diagnostics, Germany). First, paraffin-embedded sections were dewaxed, rehydrated, and then incubated in 3% H_2_O_2_ for 10 min to block endogenous peroxidase activity. Then, a mixture of TdT and dUTP was loaded to the slices and cultured at 37° C for 120 min. Added diaminobenzidine (DAB) chromogenic solution to the tissue sections and reverse stained with hematoxylin for 3 min. In the end, the slices were dehydrated by gradient ethanol (70%, 80%, 95%, and 100%), then dehydrated by gradient xylene and sealed with resin fixator. All sections were captured under a microscopy and processed by the Image-Pro Plus 6.0 software.

### Animal model

A total of 10 male nude mice were used for tumor formation experiment. As-PC cells were collected and re-suspended in medium (excluding serum) at a density of 5×10^7^ cells/mL. AsPC-1 cells stably transduced with sh-HCG11 or shRNA NC were inoculated subcutaneously at the right side of per mouse. The tumor size was measured and recorded every 7 days. After 28 days of incubation, the mice were euthanized and tumors were collected to measure their size and weight. Tumor volume was calculated by (length × width^2^). Afterward, the tissues were used to do the corresponding tests. All animal studies were approved by the Animal Ethics Committee of our hospital.

### Statistical analysis

SPSS statistics 20.0 and Graphpad Prism 6.0 software were applied to process the experimental data, which presented as mean ± standard deviation. The statistical difference of two or multiple group was processed by Student's t-test or one way analysis of variance accompanied by Bonferroni's post hoc test. P value less than 0.05 was deemed as statistically significant.
